# Rapid assessment of injection practices in Cambodia, 2002

**DOI:** 10.1186/1471-2458-5-56

**Published:** 2005-06-02

**Authors:** Sirenda Vong, Joseph F Perz, Srun Sok, Seiharath Som, Susan Goldstein, Yvan Hutin, James Tulloch

**Affiliations:** 1 Division of Viral Hepatitis, Centers for Disease Control and Prevention, Atlanta, GA, USA; 2 World Health Organization Consultant; 3 Ministry of Health, Phnom Penh, Cambodia; 4 World Health Organization, Resident Advisor, India; 5 World Health Organization, Phnom Penh Office, Cambodia

## Abstract

**Background:**

Injection overuse and unsafe injection practices facilitate transmission of bloodborne pathogens such as hepatitis B virus (HBV), hepatitis C virus (HCV), and human immunodeficiency virus (HIV). Anecdotal reports of unsafe and unnecessary therapeutic injections and the high prevalence of HBV (8.0%), HCV (6.5%), and HIV (2.6%) infection in Cambodia have raised concern over injection safety. To estimate the magnitude and patterns of such practices, a rapid assessment of injection practices was conducted.

**Methods:**

We surveyed a random sample of the general population in Takeo Province and convenience samples of prescribers and injection providers in Takeo Province and Phnom Penh city regarding injection-related knowledge, attitudes, and practices. Injection providers were observed administering injections. Data were collected using standardized methods adapted from the World Health Organization safe injection assessment guidelines.

**Results:**

Among the general population sample (n = 500), the overall injection rate was 5.9 injections per person-year, with 40% of participants reporting receipt of ≥ 1 injection during the previous 6 months. Therapeutic injections, intravenous infusions, and immunizations accounted for 74%, 16% and 10% of injections, respectively. The majority (>85%) of injections were received in the private sector. All participants who recalled their last injection reported the injection was administered with a newly opened disposable syringe and needle. Prescribers (n = 60) reported that 47% of the total prescriptions they wrote included a therapeutic injection or infusion. Among injection providers (n = 60), 58% recapped the syringe after use and 13% did not dispose of the used needle and syringe appropriately. Over half (53%) of the providers reported a needlestick injury during the previous 12 months. Ninety percent of prescribers and injection providers were aware HBV, HCV, and HIV were transmitted through unsafe injection practices. Knowledge of HIV transmission through "dirty" syringes among the general population was also high (95%).

**Conclusion:**

Our data suggest that Cambodia has one of the world's highest rates of overall injection usage, despite general awareness of associated infection risks. Although there was little evidence of reuse of needles and syringes, support is needed for interventions to address injection overuse, healthcare worker safety and appropriate waste disposal.

## Background

In many developing countries and countries with economies in transition, health care injections are overused and are frequently administered in an unsafe manner [1,2]. The World Health Organization (WHO) estimates that 20 million new hepatitis B virus (HBV) infections, 2 million new hepatitis C virus (HCV) infections, and 260,000 new HIV infections are associated with unsafe injections each year worldwide [3].

Concerns have been expressed over the use of injections within the Kingdom of Cambodia (2003 United Nations population estimate 14.1 million). Anecdotal reports suggest that therapeutic injections are often unnecessary and administered in an unsafe manner, and healthcare waste is inappropriately disposed [4-6]. A high prevalence of HIV infection has been documented in Cambodia, with 2.6% of the general population infected [7]. Cambodia also has high levels of HBV and HCV infection endemicity. A 1991 community-based study indicated that 8.0% of the population was hepatitis B surface antigen positive and 6.5% was anti-HCV positive [8]. The proportion of bloodborne pathogen infections in Cambodia attributable to unsafe injections is not known.

To estimate the magnitude and patterns of unsafe injection practices in Cambodia, a rapid assessment of injection practices was conducted. The objectives of the assessment were to describe healthcare injection practices, including measures of injection frequency and injection safety, and determine knowledge, attitudes and practices related to medical injections among the general population and healthcare providers. This assessment focused mainly on the unregulated private healthcare sector, in which 70% of Cambodians seek medical care [9].

## Methods

### Overview

The assessment was conducted using methods established by WHO for the rapid assessment of injection practices [10]. As detailed in the following sections, we interviewed members of the general population, healthcare workers responsible for prescribing injections (i.e., "prescribers"), and healthcare workers responsible for administering injections (i.e., "injection providers"). All data were collected during face-to-face interviews using standardized questionnaires and forms adapted from the WHO assessment guide [10]. The general population sample was drawn from Takeo province (1998 population census 790,168), a rural province located in southeastern Cambodia near the Vietnamese border. Prescribers and injection providers were sampled in Takeo province and in Phnom Penh (1998 population census 999,809), Cambodia's largest city [11].

### General population

We surveyed 500 Takeo province residents in November and December 2002 using standard cluster sampling methodology. To obtain a total of 500 participants, we surveyed 25 participants each in the catchment area of 20 district health centers. The 20 district health centers were selected by probabilities proportional to the population size of their catchment areas [12]. For each health center selected, one village was chosen at random to be surveyed. Households were sampled at random until 25 participants were included. Verbal informed consent was obtained from all household members >16 years old to participate in the survey. For persons ≤ 16 years old, informed consent was obtained from a parent or guardian. For each participant, basic demographic data were collected, as well as the number of injections received during the 6 month period beginning on April 13, 2002 (New Year's Day in Cambodia). Injections were categorized as therapeutic injections, immunizations, and intravenous infusions. Participants >16 years of age were also interviewed regarding their knowledge and attitudes about injections.

### Prescribers and injection providers

We surveyed prescribers and injection providers in Takeo province and Phnom Penh in November and December 2002. In Takeo province, a convenience sample of 30 prescribers and 30 injection providers was accessed through the province's five public hospitals. In Phnom Penh, 30 prescribers were randomly selected from a list of registered private outpatient clinics and 30 injection providers were selected from 11 of the city's 13 private hospitals. For all prescribers, regardless of where they were identified, the survey focused on their medical practices in their private outpatient clinics. We ascertained their knowledge, attitudes and practices regarding injections. Injection frequency was determined by asking prescribers to estimate the total number of prescriptions they wrote per week and the number that included an injection or infusion. Injection providers were interviewed regarding their knowledge of disease transmission risks through unsafe injections, their hepatitis B vaccination status and the frequency of needle stick injuries during the previous 12 months. In addition, we observed each injection provider administer one injection. During all hospital visits, information was recorded regarding the use of incinerators and the presence of used needles and syringes and other medical equipment on the grounds of the facility.

### Data analysis

Proportions, means, and confidence intervals were calculated using EpiInfo 6.04d (CDC, Atlanta, Georgia) with adjustment for the cluster sampling design effect. Univariate and multivariate analyses of variables associated with receiving injections were performed using SAS software version 8 (SAS Institute, Cary, North Carolina).

## Results

### General population

The overall injection rate was 5.9 injections per person-year (95% CI: 5.3 – 6.7), with 40% of the participants reporting receipt of one or more injections during the previous 6 months (Table 1). Therapeutic injections accounted for 74% of the total number of injections reported, followed by intravenous infusions (16%), and immunization (10%). Among individuals who had any exposure to injections, nearly one-half reported receiving ≥ 5 injections. This group represented only 18% of all participants but accounted for 69% of the total injections reported.

**Table 1:** Characteristics of study participants, Takeo province, Cambodia, 2002

	**Total (n = 500)**	Children* (n = 212)	**Adult women (n = 189)**	**Adult men (n = 99)**
	
**Age:**				
median	22	9	38	37
range (years)	0–80	0–16	17–79	17–80
***Injection experiences (6-month period)***				
Number of injections	1483	557	713	213
Overall injection rate (per person-year)	5.9	5.3	7.5	4.3
Proportions of subjects who received:				
≥ 1 vaccine injection	15%	35%	0%	0%
≥ 1 therapeutic injection	32%	31%	39%	20%
≥ 1 intravenous infusion	16%	16%	18%	14%
≥ 1 injection (any type)	40%	46%	42%	24%
≥ 5 injections (any type)	18%	18%	22%	9%
***Knowledge and attitudes^***				
Trusted practitioners if no injections prescribed	90%	ND	90%	89%
Preferred injection for treatment of fever	32%	ND	35%	26%
Believed injections more powerful than oral medication	47%	ND	50%	40%
Aware that dirty syringes can transmit HIV	95%	ND	96%	93%
Aware that dirty syringes can transmit hepatitis	59%	ND	60%	57%

ND = not determined

* participants aged ≤ 16 years

^ adults only (288 total respondents)

The pattern of injection use differed by age and sex. Among adults, women were more likely than men to report receiving any (42% vs. 28%, p < 0.001) or frequent injections (i.e., ≥ 5 injections during the previous 6 months; 22% vs. 9%, p < 0.001) (Table 1). Women were also more likely than men to report having received ≥ 1 therapeutic injection (39% vs. 20%, p = 0.001). Similar proportions of men, women and children reported receiving intravenous infusions, ranging from 14 – 18%. Immunizations were limited to children, with 35% of children reporting having received ≥ 1 immunization.

**Figure 1:**
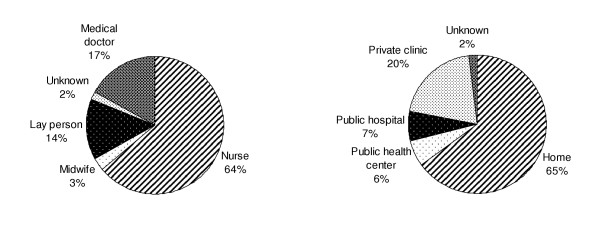
Distribution of injections by provider type and setting*, Takeo Province, Cambodia, 2002 (n = 96) *Data refer to the most recent injection received by adults reporting ≥ 1 injection in the previous 6 months

Ninety-six adult participants (93% of those reporting one or more injection in the previous 6 months) could recall the details of their last injection. All reported the injection was administered with a newly opened disposable syringe and needle. The majority (85%) of these injections was administered by health care workers; at least 13% were administered by lay persons (Figure 1). Injections were most commonly administered in the patient's home (65%) or in a private clinic (20%). Only 13% of injections were administered at public hospitals or public health centers.

Of the 288 subjects interviewed, 90% reported they would trust a medical practitioner who did not prescribe an injection (Table 1). However, 47% (134/288) believed that therapeutic injections are more powerful than oral medications, with 75% (100/134) of these persons attributing a faster healing effect to injections. In addition, 32% of the respondents reported they preferred injections over oral medications for the treatment of illnesses characterized by fever. Knowledge of the HIV transmission risk associated with "dirty" syringes and needles was very high (95%), though only 59% of respondents were aware that hepatitis could be transmitted in this manner. None of the knowledge or attitude variables or other characteristics (besides gender) were associated with the receipt of injections. This was true even when the analyses were restricted to comparisons of persons reporting no injections versus frequent injection use (i.e., ≥ 5 injections during the previous 6 months).

### Prescribers

The 60 prescribers interviewed were registered medical doctors or medical assistants. Prescribers estimated that nearly half (47%) of the total prescriptions they wrote included either a therapeutic injection (34%) or an intravenous infusion (14%) (Table 2). The overall prescription rates and distribution by type was similar in Phnom Penh and Takeo Province. The main reasons cited for prescribing injections were illness severity (44%) and perceived patient preferences for injectable medications (40%). Seventy-seven percent of prescribers believed that injection prescriptions resulted in greater reimbursement. Only one prescriber perceived himself as over-prescribing injections. Most (92%) prescribers were aware that HIV, HBV and HCV could be transmitted through unsafe injections (Table 2).

**Table 2:** Characteristics of injection prescribers and providers, Takeo province, Cambodia, 2002

	**Total (n = 60)**	**Takeo Province (n = 30)**	**Phnom Penh (n = 30)**
	
***Characteristics of prescribers^1^***			
Medication prescription rate (prescriptions/week)			
average	20	21	20
median	20	21	20
range	4–140	4–140	7–140
			
Prescriptions including an injection^2^	47%	48%	45%
therapeutic injection	34%	32%	35%
intravenous infusion	14%	18%	10%
			
Main reason for prescribing injections			
illness severity	44%	50%	37%
patient preference	40%	40%	40%
more effective than oral medications	12%	7%	17%
reimbursement	9%	7%	10%
			
Preferred injectable med for treatment of febrile illness	64%	47%	80%
Believed patient trust requires injection prescription	42%	53%	30%
Believed reimbursement is higher for patient visits that result in injection prescription	77%	87%	66%
Perceived themselves as over-prescribing injections	2%	3%	0%
Knew HIV, HBV and HCV can be transmitted through unsafe injections	92%	87%	97%
			
***Characteristics of injection providers***			
Completed hepatitis B vaccination series	20%	7%	33%
Needlestick injury in last 12 months	53%	50%	57%
Average number (and range) of needlesticks in past 12 months among those reporting one or more	1.7 (1–10)	1.4 (1–6)	2.0 (1–10)
Use of single use needles and syringes^3^	98%	97%	100%
Safety box (i.e., sharp container) present in injection administration area^3^	25%	37%	13%
Reported having sufficient number of sharps boxes	85%	77%	93%
Practiced two hand recapping of used needles^3^	58%	53%	60%
Left used sharps in preparation area^3^	13%	23%	3%
Knew HIV, HBV and HCV can be transmitted through unsafe injections	90%	87%	93%

^1 ^Prescribers' responses pertain to their private outpatient practices

^2 ^Denominator is the total number of weekly prescriptions

^3 ^Based on observation of provider by the investigators

### Injection providers

All 60 injection providers interviewed were registered nurses, only 20% of whom had received the complete hepatitis B vaccination series. During our observations, 59 (98%) providers used new single use syringes (i.e., traditional plastic disposable syringes) and needles and one administered the injection with sterilized reusable equipment. Over half (58%) of the providers were observed practicing two-handed recapping of used injection equipment and 53% reported they had sustained a needlestick injury during the past 12 months. For only 25% of providers, a safety box (i.e. sharps container) was observed in close proximity to the injection area, yet 85% reported having a sufficient number of safety boxes. We observed that 13% of providers left used sharps in the injection preparation area (23% in Takeo province and 3% in Phnom Penh city). Ninety percent of injection providers stated they were aware that HIV, HBV and HCV could be transmitted through unsafe injections (Table 2).

### Waste disposal

In the general population survey in Takeo province, 32% (23/72) of participants who reported receiving their last injection in their home reported that the used injection equipment was left behind by the injection provider. Used sharps were observed on the grounds outside three of the five public hospitals in Takeo province, but not on the grounds of any of the 11 private hospitals in Phnom Penh. Incinerators and sharps pits in which used sharps waste could be burned or buried were present at all of the Takeo hospitals. In Phnom Penh, sharps pits were available in two hospitals. In the remaining nine hospitals, sharps waste was collected along with non-medical waste for disposal at the city landfill.

## Discussion

The rapid assessment of injection practices described in this paper suggests that injections are prescribed and administered in Cambodia at excessive rates. Intravenous infusions were common among the general population surveyed and represented approximately one-third of all injections prescribed in private outpatient clinics. Using WHO standardized measures as a basis for comparison, our assessment indicates that Cambodia has one of the highest rates of overall therapeutic injection usage ever reported worldwide [1,2]. Studies conducted in other countries in the region have documented rates of 2–4 injections/person/year; approximately one third lower than in Cambodia [2].

This assessment documented potentially harmful injection practices including inadequate handling and disposal of used injection equipment. Given the country's high prevalence of HIV, HBV, and HCV, the overuse and misuse of injections carry substantial risks. While reuse of injection equipment was uncommon in this survey, the high prevalences of bloodborne infections may reflect past practices of needle and syringe reuse. We did observe breaks in aseptic technique (e.g., used sharps left in the injection preparation area) that may facilitate cross-contamination during injection preparation [13,14]. Other infection control breaks such as mishandling of sterile materials and improper use of multidose vials have been frequently implicated in the transmission of bloodborne viral and bacterial infections, but were not studied in this survey [13,15]. Anecdotal reports suggest that used needles and syringes might be repackaged and resold as new. While there was no evidence to suggest this practice was occurring in Cambodia, we did not evaluate the sterility of needles and syringes. In addition, many of the injection providers in our survey engaged in high risk behaviors (e.g., two-handed recapping needle), highlighting the need for training and other interventions aimed at reducing occupational exposures and infection risks.

The patterns of attitudes, knowledge and practices regarding injections among the general population and health professionals observed in Cambodia were similar to those in many countries with high frequencies of injections and comparable healthcare systems such as Pakistan, India, and Indonesia [16-18]. In these countries as in Cambodia, patients and prescribers frequently believe that injections are more effective than oral treatments, and few health professionals perceive themselves as over-prescribing injections. This study and others have also shown that many healthcare providers believed that patients' trust hinged on injection prescriptions, whereas only a minority of patients actually stated that they prefer injections for treatment of conditions that could be treated with oral medications. Both misconceptions may facilitate injection overuse.

In this study, almost half of the prescriptions made by the private providers we surveyed contained at least one injection, compared with a previous finding that only 2% of prescriptions written in public sector outpatient clinics included injections [19]. Financial interests might lead some private health professionals to over-prescribe injectable medications [16]. Since oral and intravenous medications are readily available directly from pharmacies without prescriptions, it is possible that private healthcare providers provide and promote injections and infusions as a way to attract patients [20]. These factors might partly explain the popularity of the private sector compared with the public healthcare sector where the cost for clinic visits is lower and medications are available free of charge, but are dispensed according to standardized treatment protocols which generally specify oral formulations.

High levels of awareness of infection risks associated with syringe and needle reuse, particularly regarding HIV were documented in this study. This is consistent with results of a recent national Demographic Health Survey [9] and likely reflects Cambodia's efforts during the late 1990's to educate the general public regarding risk factors for HIV infection. These education campaigns included information on the risk of transmission of HIV from re-used syringes. Following the campaigns, disposable needles and syringes became widely available in Cambodia at a relatively low cost. It is possible that the increased awareness of HIV infection risks associated with injections led to increased utilization of disposable needles and syringes rather than a shift toward alternative forms of treatment.

This assessment had several limitations. First, the survey did not capture the medical indications that were associated with either prescription or receipt of injections. Second, resource constraints required the general population surveys to focus on one province instead of being conducted nationwide. Takeo province was selected because it is the third most populated province, its economic status is in the middle of the national spectrum, and like the general Cambodian population it is mainly rural [9]. Third, because only formal health professionals were surveyed, we could not assess practices in the informal health sector. We found that approximately one-in-seven injections in Takeo province were administered by untrained paramedical and lay health care workers and their practices may differ from health care workers in the formal sector.

There is growing consensus that improvements in injection safety and overuse in developing countries can be achieved [21]. Such efforts require multidisciplinary engagement and behavioral changes on the parts of both patients and health professionals, including those from the private healthcare sector [22]. Although large-scale interventions to improve the safety of therapeutic injections are limited, several demonstration projects have proven to be effective and relatively long lasting. Studies in Indonesia demonstrated a sustained 19% reduction in injection rates following "interactional group discussions," an intervention that brings health care providers and patients together to discuss their preferences regarding injections and oral medications [18,23]. In Burkina Faso, interventions targeted at providing sufficient quantities of single-use syringes and needles resulted in >90% reductions in unsafe injections that have been sustained for at least 5 years [24].

Comprehensive interventions aimed at reducing overuse of injections, promoting rational use of oral and injectable medications, and reinforcing safe injection and waste disposal practices are likely to be cost-effective in Cambodia [25,26]. While initial efforts in injection safety have been largely focused on infant immunization, Cambodia is now expanding this initiative to include curative care, with an emphasis on the private sector. A national plan of action has been developed to decrease injection use and promote injection safety in accordance with WHO guidelines. The plan aims to involve multidisciplinary partners and improve coordination among relevant departments in the Ministry of Health. This plan also includes demonstration projects that address specific issues related to injection use in the private sector. It is hoped that Cambodia's efforts to address its injection safety and overuse problems will provide an example for other countries that are beginning to confront this important public health issue.

## Competing interests

The author(s) declare that they have no competing interests.

## Authors' contributions

SV, SS, SS and JT adapted the WHO assessment design to Cambodia. SV, SS, and SS conducted the survey, supervised different aspects of its implementation and collected data. SV and JP synthesized analyses and led the writing. SG helped interpret findings and review drafts of the manuscript. YH conceptualized the assessment and reviewed drafts of the manuscript. All authors read and approved the final manuscript.

## Pre-publication history

The pre-publication history for this paper can be accessed here:


